# Vision as oculomotor reward: cognitive contributions to the dynamic control of saccadic eye movements

**DOI:** 10.1007/s11571-020-09661-y

**Published:** 2021-01-25

**Authors:** Christian Wolf, Markus Lappe

**Affiliations:** Institute for Psychology, University of Muenster, Fliednerstrasse 21, 48149 Münster, Germany

**Keywords:** Eye movement, Saccade, Reward, Vision, Saccadic adaptation, Oculomotor control

## Abstract

Humans and other primates are equipped with a foveated visual system. As a consequence, we reorient our fovea to objects and targets in the visual field that are conspicuous or that we consider relevant or worth looking at. These reorientations are achieved by means of saccadic eye movements. Where we saccade to depends on various low-level factors such as a targets’ luminance but also crucially on high-level factors like the expected reward or a targets’ relevance for perception and subsequent behavior. Here, we review recent findings how the control of saccadic eye movements is influenced by higher-level cognitive processes. We first describe the pathways by which cognitive contributions can influence the neural oculomotor circuit. Second, we summarize what saccade parameters reveal about cognitive mechanisms, particularly saccade latencies, saccade kinematics and changes in saccade gain. Finally, we review findings on what renders a saccade target valuable, as reflected in oculomotor behavior. We emphasize that foveal vision of the target after the saccade can constitute an internal reward for the visual system and that this is reflected in oculomotor dynamics that serve to quickly and accurately provide detailed foveal vision of relevant targets in the visual field.

## Introduction

The visual system of humans and other primates is characterized by a clear division of labor: Whereas the central part of the visual field, the fovea, can process fine visual details, peripheral acuity declines rapidly with increasing eccentricity. The differences between foveal and peripheral processing start in the retina with the different photoreceptors and their density, and the differences are continued and intensified along the visual hierarchy (for reviews see Strasburger et al. 2011; Yu et al. 2015; Stewart et al. 2020). As a consequence of such a foveated visual system, the fovea must be reoriented towards objects or regions in the visual field that were selected using the periphery and considered relevant or interesting for further visual inspection. This re-orientation of the fovea is achieved by means of saccadic eye movements, quick movements of the eyeball which, depending on their amplitude, last between 20 and 80 ms and saturate at an angular velocity of around 500 deg/s (Bahill et al. 1975; Collewijn et al. 1988; Gibaldi and Sabatini 2020).

In everyday life we make about three saccades per second. Although saccades subjectively require little if no cognitive effort, the saccade system has become a role model to study cognitive processes like attention, learning and decision making. Its advantages are that the sensory input can be easily controlled, the motor output can be directly and objectively measured, and the saccade network includes almost all major anatomical structures of the brain. Or as Carpenter (1994) has phrased it: The saccade system is a “microcosm of the brain”. Carpenter (1981) also noted that two things are very striking about the reaction time of saccadic eye movements: they are surprisingly long, and they are surprisingly variable. The shortest possible route from sensory input to motor output, thus from the retina via the superior colliculus (SC) to the brainstem and the extraocular muscles, would take approximately 60 ms of signal transduction. However, the eyes often require a three- or fourfold of this time before they actually start to move. Carpenter (1981; see also Noorani and Carpenter 2016) remarked that the pathway through the superior colliculus would do a satisfying job if all the oculomotor system had to do was to translate a visual signal into a motor response. Yet, the neurons in the superior colliculus would only know *where* the target is, but not *what* it is. Determining the identity of a target could only be achieved in higher cortical areas of the oculomotor network. These cortical sites provide direct excitatory input to the SC that sets up the target for the eye movement as well as indirect inhibitory input to the SC via the basal ganglia. The latter prevent the superior colliculus from responding too early until a better analysis of what to look at is carried out (for review see Hikosaka et al. 2000). Carpenter (1981) thus remarked that saccadic eye movements can be considered a decision not only in space but also in time, because saccade latencies are prone to “oculomotor procrastination” and that most of the reaction time is decision time used to arrive at a more sophisticated decision of *what* to look at (Carpenter 1981).

Here, we review evidence that saccadic eye movements are sensitive to cognitive processes and their sensory consequences: In the first part, we briefly sketch the neural oculomotor circuitry and how behavioral relevance and valuation processes manifest themselves in neural activity. In the second part, we provide a short overview which oculomotor parameters can be used to measure higher-level influences and what these metrics reveal about the underlying mechanisms. We specifically focus on saccade latencies, the kinematics of the movement itself and the maintenance of saccade accuracy. In the third part, we review how eye movement control is modulated by reward, task-relevance and image content, using these metrics as probes into the system. We emphasize that vision of the target after the saccade can be a rewarding process itself and that this is reflected oculomotor behavior.

While this review focusses explicitly on saccades, it is important to note that other eye movements like microsaccades and smooth pursuit also show cognitive influences. Moreover, saccades in turn also influence cognitive processes like memory or visual attention and perception. For these and other topics, we refer the reader to several excellent review articles (Gegenfurtner 2016; Hutton 2008; Rolfs 2009; Rucci and Poletti 2015; Schütz et al. 2011; Spering and Montagnini 2011; Tatler et al. 2011).

### Neural circuitry of saccade control

The neural circuit controlling saccadic eye movements receives sensory input from the visual system, but it does more than just translating this sensory input into a motor response. Interposed between motor output and sensory input is a neural architecture mainly devoted to target selection mechanisms. Here, we briefly sketch the oculomotor circuit with a special emphasis on those sites sensitive to high-level processes and which code the behavioral relevance of a target or are involved in valuation and learning. For a more complete review of the neural oculomotor circuit see (Krauzlis 2005; Leigh and Zee 2015; Munoz and Everling 2004; White and Munoz 2011).

At the end of the oculomotor pathway are the three pairs of extraocular muscles which control all rotations of the eye and receive their input from a circuit in the brainstem which is said to act as a burst generator (Robinson 1975; Scudder 1988; for reviews see Scudder et al. 2002; Sparks 2002). This circuit controlling the transition between periods of fixation and saccades in turn receives direct input from the superior colliculus (SC). The SC is a midbrain structure at the center of the oculomotor network that is crucial for orienting responses but also plays an important role in covert attention, multisensory integration and decision making (for reviews see Basso and May 2017; Krauzlis et al. 2013; Stein and Stanford 2008; White and Munoz 2011). The superficial layers of the SC (SCs) mainly receive visual input from two sources, directly from the retina as well as from visual cortex. The intermediate layers on the other hand (SCi) receive widespread input from cortical and subcortical regions. The input sites include, for example, frontal cortex (frontal eye fields, FEF; supplementary eye fields, SEF), parietal cortex (lateral intraparietal area, LIP) as well as the basal ganglia. As a consequence, neurons in SCs can be characterized as purely visual, whereas neurons in SCi integrate motor signals, signals from different sensory modalities as well as cognitive signals. The SCi can thus be considered a final junction for the control of saccades.

The lateral intraparietal area (LIP) in the parietal cortex receives input from many visual areas, has interconnections with frontal areas and projects to the intermediate layers of the superior colliculus (Baizer et al. 1991; Blatt et al. 1990; Ferraina et al. 2002; Lewis and van Essen 2000; Paré and Wurtz 2001). The visual responses of neurons in LIP are different from the visual responses in lower visual areas in a way that they do not simply reflect the low-level properties of the stimulus but also whether the stimulus is behaviorally relevant or not. Gottlieb et al. (1998) showed that the firing rate of LIP neurons is higher if a task-irrelevant stimulus suddenly appears inside their receptive field compared to a continuously displayed stimulus that was brought inside the receptive field by means of a saccade. The same modulation in firing rate was observed when the target was behaviorally relevant (Gottlieb et al. 1998) or signaled the availability of reward (Bendiksby and Platt 2006; Sugrue et al. 2004). Traditionally, the posterior parietal cortex was associated with either movement intention (Andersen and Buneo 2002) or spatial attention (Colby and Goldberg 1999). More recently however, the viewpoint emerged that particularly LIP acts as an integrated map combining visual, cognitive and motor signals in order to code the priority of targets (Bisley and Goldberg 2010; Gottlieb 2007; Gottlieb et al. 1998; Ipata et al. 2009).

The concept of a priority map is based on the saliency-map model (Itti and Koch 2000). The saliency map is a theoretical two-dimensional representation of space that describes how bottom-up visual inputs guide eye movements or visual attention: Visual images are first pre-attentively filtered into different feature maps (orientation, color, luminance) before the activity in the feature maps is linearly combined into a single saliency map. The location with the highest activity in the map is selected as the subsequent target using a winner-take-all mechanism (Itti and Koch 2000). The priority map extends this concept by combining bottom-up information about salience with top-down information about the behavioral relevance of a target (Bisley and Mirpour 2019; Fecteau and Munoz 2006; Serences and Yantis 2006). In addition to LIP, the SCi (White and Munoz 2011) as well as the frontal eye field (Thompson and Bichot 2005) have been characterized as a priority map.

The frontal eye field (FEF) projects to the brainstem via the SC and also via a direct projection to the burst generator. Stimulation in FEF elicits contralateral saccades (Robinson and Fuchs 1969). However, the direct projection to the burst generator has been described as insufficient in generating saccades when the SC was pharmacologically inactivated (Hanes and Wurtz 2001), suggesting that the signal triggering the saccade reaches the brainstem over pathways through the SC. In humans, the contribution of the FEF to saccade initiation is for example shown by the relationship between premotor activity in FEF and saccadic reaction times (Connolly et al. 2005). Within the FEF, conflicting saccade vectors are inhibited preceding saccade initiation (Schlag et al. 1998) and stimulation in FEF excites the same but inhibits different saccade vectors in SC (Schlag-Rey et al. 1992), highlighting that activity in FEF is not only relevant for selecting a target but also plays a crucial role in distractor suppression (Cosman et al. 2018). Activity in FEF can reflect value (Roesch and Olson 2003) and the FEF plays a role in decision making as well as in evaluating preceding choices (Ding and Gold 2012; Teichert et al. 2014), possibly for the optimization of future behavior. FEF can modify visual responses in area V4 (Moore and Armstrong 2003) which could be attributed to dopaminergic activity (Noudoost and Moore 2011).

Two additional sites that play an important role in the oculomotor circuit are the cerebellum and the basal ganglia. Both sites have recurrent connections to cortical regions and play a profound role in motor control and learning which becomes apparent from the motor deficits associated with lesions in either site. Although the cerebellum is also involved in cognitive tasks (for reviews see De Smet et al. 2013; Schmahmann et al. 2019), it is of particular importance for error-based learning (Doya 2000) and thus for the maintenance of accuracy in saccade adaptation paradigms (Optican and Robinson 1980; for reviews see Pélisson et al. 2010; Soetedjo et al. 2019; Thier and Markanday 2019). Given the small size of the fovea of around 1 degree visual angle, saccades must be accurate to provide high-acuity vision of the target. Moreover, visual sensitivity is reduced during saccades (Bridgeman et al. 1975; Burr et al. 1994; Dodge 1905) and the need for corrective saccades would further interrupt visual processing. In the laboratory, the maintenance of saccade accuracy is typically studied by consistently displacing the target during the saccade (McLaughlin 1967). These displacements give rise to a gradual adjustment of saccades and a reduction of the visual error which is achieved by an interplay of simple spike and complex spike activity by Purkinje cells located in the oculomotor vermis of the cerebellum. The simple-spike activity of Purkinje cells shows a population response that encodes the duration (Thier et al. 2000; Catz et al. 2008) and velocity profile (Herzfeld et al. 2015) of saccades by which, in turn, the saccade gain is controlled. Whereas these simple spikes occur with a high frequency, the less frequent complex spikes encode the experienced error, with the probability and temporal distribution of complex spikes being related to error direction and magnitude respectively. These complex spikes correspondingly change simple spike activity and the saccadic movement in the subsequent trial (Herzfeld et al. 2018). This error signal originates in the superior colliculus which changes complex spike activity and thus saccade adaptation via projections to the inferior olive (Kojima and Soetedjo 2018).

The basal ganglia consist of several subcortical nuclei of which two have a clearly defined role in the oculomotor circuit: the substantia nigra pars reticulate (SNr) and the caudate nucleus (CD). The SNr projects to the SCi and provides sustained inhibitory input. In turn, the SNr can receive inhibitory input from the CD which results in disinhibition and thus excitation of the SC (for review see Hikosaka et al. 2000). Besides this direct CD-SNr-SCi pathway, additional indirect and hyperdirect pathways serve the possible enhancement of inhibition (for review see Nambu et al. 2002), highlighting that the basal ganglia strongly influence which option is selected and which option is suppressed by modulating the level of inhibition.

But how does the caudate nucleus decide which target to select? Findings from the last decade have shaped the view that the CD-SNr-SCi circuit might be composed of parallel pathways that code the short-term and the long-term value of targets respectively (Kim and Hikosaka 2013, 2015; for review see Hikosaka et al. 2014). Neurons in the head of the caudate (CDh) mainly receive input from frontal areas and are sensitive to value that is flexible and varies on a short-term basis. In contrast to that, the tail of the caudate that mainly receives input from temporal areas and codes value that has been acquired on a long-term basis. Neurons in the body of the caudate, that are positioned in-between head and tail respond to both, short-term and long-term value, suggesting that the caudate represents a gradient from short-term to long-term value learning rather than two distinct mechanisms. Yet, head and tail of the caudate project to different subregions of the SNr whose outputs converge in the superior colliculus.

Taken together, multiple signals converge in the superior colliculus: bottom-up visual information, top-down information inherited from frontal and/or parietal regions as well as short-term and long-term value information from the dopaminergic midbrain. These various signals have their own temporal dynamics (Siegel et al. 2015) and can be reflected in the command that generates the saccade as well as the dynamic control of the movement itself (see below). Although the cerebellum is crucial for oculomotor learning and thus for the maintenance of saccade accuracy, recent evidence suggest that the error signal driving saccade adaptation is provided by the superior colliculus. This allows the contribution of other signals beyond a purely visual error to participate in the maintenance of saccade accuracy.

### What’s in an eye movement?

Every saccadic eye movement provides a manifold of information. Most of its temporal and spatial characteristics, as well as the dynamics of the movement itself, are sensitive to cognitive modulations. We provide a short overview which aspects of saccadic eye movements can be used to measure cognitive influences and what these metrics reveal about the underlying mechanisms.

#### Temporal: when the eyes move

How an eye movement is temporally characterized depends on whether the saccade is initiated voluntarily or as a response to an external event like the appearance of a stimulus. In the former case, for example when reading a book or inspecting a photograph, eye movement behavior is temporally characterized by the time between two eye movements: the fixation duration (sometimes also referred to as dwell time). How long a fixation will last also depends on what is currently inspected with the fovea. In reading, fixation durations depend on non-lexical factors like word-length (Kliegl et al. 2004; Rayner and McConki, 1976) and lexical factors like the predictability of a word in a given context (Ehrlich and Rayner 1981; for review see Staub 2015). Similarly, fixation durations in scene viewing are longer when inspecting objects that are unexpected given the scene content (Henderson et al. 1999; Loftus and Mackworth 1978; Võ and Henderson 2009). Studies on natural scene viewing have further shown that fixation durations can depend on the current task (Nuthmann 2017; Nuthmann et al. 2010; Võ and Henderson 2009) as well as on the competition between the current foveal input and potential saccade targets inspected in the periphery (Einhäuser et al. 2020; Laubrock et al. 2013; Tatler et al. 2017).

**Fig. 1:**
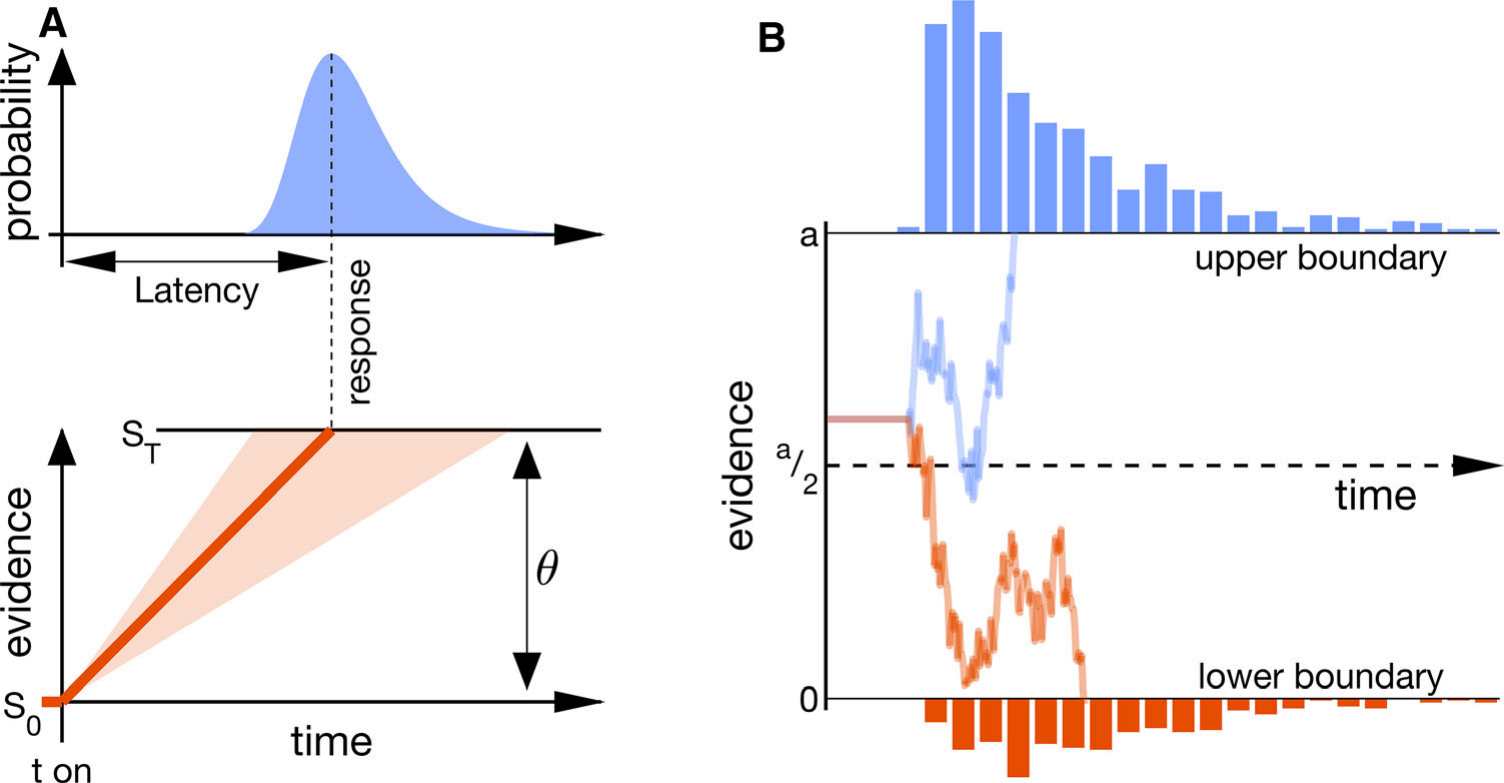
Rise-to-threshold models for the analysis of saccade latencies. The panels show, at the bottom, evidence accumulation as a function of time and, on top, the resulting latency distribution(s). **a** LATER model in its simple form. Upon target onset (t on), the evidence starts at baseline, *S*_*O*_, and accumulates until the saccade threshold, *S*_*T*_, is reached and the saccade is initiated. The orange line depicts an exemplary rate-of-rise of the evidence signal. The rate-of-rise is considered constant within every trial but can vary from trial to trial (shaded orange region), giving rise to the typical shape of a latency distribution (blue). The model comprises four parameters: baseline level, threshold, the mean rate-of-rise, and its variability. Without physiological data, it is only possible to infer the distance between baseline level and threshold, but not to attribute changes to either of the two. The model can be extended to include, for example, inhibition among potential future saccade targets (Leach and Carpenter 2001; Noorani and Carpenter 2015) or among potential saccade targets and the current gaze position (Story and Carpenter 2009; Tatler et al. 2017) (**b**) Drift–diffusion model. The drift–diffusion model describes noisy evidence accumulation over time in a binary choice scenario (for multi-alternative diffusion-modelling see Krajbich and Rangel 2011). The decision signal starts somewhere in-between two boundaries and accumulates evidence until one of these boundaries, $a$ or $0$, is reached. Unlike LATER, the rate of evidence accumulation, the drift rate, is variable within a trial. Thus, even when the mean drift rate favors one options, the other option can ultimately be selected due to the noise in the decision process. Traces represent evidence accumulation in trials with median reaction time for each response option respectively. In this example, evidence accumulation starts above $a/2$, representing an initial bias in the decision. A bias towards one option can be observed when one of the two options is a priori more likely or associated with a higher payoff (Leite and Ratcliff 2011; Mulder et al. 2012). If the boundary separation, $a$, is small rather than large, responses occur earlier but will be more error-prone. The boundary distance, $a$, therefore characterizes the trade-off between speed and accuracy (Milosavljevic et al. 2010; Palmer et al. 2005; Ratcliff and Rouder 1998). In addition to the standard parameters (boundary separation, bias, drift rate and variability), the model is often extended to include a non-decision time, and its variability (Ratcliff and Tuerlinckx 2002) as well as variability in the starting point. The flat horizontal part at the beginning of the two traces denotes the non-decision time

In laboratory studies, saccades are often made in response to an appearing stimulus and the saccadic reaction time, the saccade latency, is expressed as the temporal difference between target onset and saccade onset. Saccadic reaction times can be successfully described by rise-to-threshold mechanisms (Fig. 1): an evidence signal is accumulated until a threshold is reached and the saccade is carried out. Although measuring distributions and analyzing them with a rise-to-threshold model requires more data (Lerche et al. 2017) and is more time-consuming than just comparing mean values of saccade latencies and choice, it is often worth the effort, because these models are able to distinguish processes that might give rise to indistinguishable behavioral results (Lerche and Voss 2020). Two successful applications to distributions of saccadic reaction times are the LATER model (Linear Approach to Threshold with Ergodic Rate; Carpenter 1981; for reviews see Noorani 2014; Noorani and Carpenter 2016) and drift–diffusion modelling (DDM; Ratcliff 1978; Ratcliff et al. 2016; Vandekerckhove and Tuerlinckx 2007). Whereas LATER has traditionally been used to study saccade selection, diffusion modelling has found many implementations in all fields of decision making. The decision which model to use will depend on the research question or the research design at hand. LATER is attractive because of its conceptual simplicity and the few parameters it has, whereas the drift–diffusion model provides more degrees of freedom and its usage is facilitated by several freely available toolboxes (Vandekerckhove and Tuerlinckx 2008; Voss et al. 2015; Wiecki et al. 2013).

Both models can account for the systematic effects of changing low-level properties of the target as well as changing the constraints of the task or the state of the participant (Carpenter 2004; Carpenter and Williams 1995; Milosavljevic et al. 2010; Palmer et al. 2005; Reddi and Carpenter 2000). Moreover, both models qualify for the common analysis of saccade latencies and errors in tasks in which these two variables are the main measurement of interest. For example, in the anti-saccade and the Go/No-go task, two oculomotor tasks often used by clinicians to probe response inhibition (Antoniades et al. 2013; Gomez et al. 2007; Hutton 2008; Noorani and Carpenter 2013). Either model allows to identify impairments in the underlying cognitive processes in diseases like ADHD or Parkinson’s disease rather than just to describe the behavioral performance (Huang-Pollock et al. 2017; Michell et al. 2006; Zhang et al. 2016).

#### Saccade kinematics: how the eyes move

Every saccadic eye movement is the result of precise acceleration and deceleration of the eyeball resulting in a unimodal and mostly symmetrical velocity profile. Whereas the peak-velocity of saccades can vary strongly across individuals (Boghen et al. 1974; Reppert et al. 2018) and between saccade directions (Collewijn et al. 1988; Vergilino-Perez et al. 2012), peak-velocity as well as the saccade’s duration are mainly determined by one factor: saccade amplitude. The relationship between saccade amplitude on the one hand and peak-velocity and duration on the other hand is referred to as main sequence (Bahill et al. 1975; Gibaldi and Sabatini 2020): For amplitudes up to around 20 degrees visual angle, peak-velocity and duration increase linearly with amplitude, whereas peak-velocities start to saturate for larger amplitudes. Therefore, when comparing peak-velocities between two conditions one has to bear in mind that changes in peak-velocities might be caused by changes in saccade amplitude. Two possibilities to account for the amplitude dependence would be to either measure a whole range of amplitudes and compare the whole main sequence or to compute a velocity index that is independent of saccade amplitude (e.g. Lebedev et al. 1996).

It recently became clear that despite their strong dependence on saccade amplitude, peak-velocities are additionally sensitive to motivation (e.g. Muhammed et al. 2020), valuation (e.g. Reppert et al. 2015) and arousal (for review see Di Stasi et al. 2013). Due to its relationship with arousal, peak-velocity has in turn become a marker for fatigue, both in clinical settings (Ferreira et al. 2017; Finke et al. 2012) as well as in the field of cognitive ergonomics (Di Stasi et al. 2014; Hirvonen et al. 2010). A further frequently reported kinematic marker of saccades is the saccade trajectory that reflects the temporal dynamics of attraction and inhibition in the oculomotor system and has been comprehensively reviewed (for reviews see van der Stigchel 2010; van der Stigchel et al. 2006).

#### Spatial: keeping eye movements accurate

Visual contrast sensitivity (Burr et al. 1994) as well as sensitivity to image displacement are drastically reduced during saccadic eye movements (Bridgeman et al. 1975). As a consequence, we do not become aware of some changes to the visual world that occur during saccades. This can easily be demonstrated by standing close to a mirror, looking at one of your eyes and then shifting your gaze from one eye to the other. You will miss seeing most of your own eye movement. (However, things will be different if you use a front-facing camera on a mobile device instead of a mirror. Here, you can actually see your eyes move because of the temporal lag). Likewise, a slight displacement of the target during the saccade usually escapes the awareness of the participant. Moreover, such a displacement cannot be compensated online (i.e. while the eyes are in flight), because latencies of visual processing are longer than usual saccade durations. The result is a mismatch between the actual saccade endpoint and the point where the saccade was aimed. The maintenance of saccade accuracy therefore has to occur from one trial to the next. Only accurate saccades guarantee high-acuity processing of the saccade target and reduce the requirement of further corrective saccades that would in turn again interrupt visual processing. If the target is consistently displaced in the direction of the saccade, the oculomotor system increases saccade amplitude over the course of the experiment. Conversely, amplitudes shorten when the target is stepped backward. This dynamic adjustment of saccade amplitude is referred to as saccade adaptation and is typically studied using the double-step paradigm (McLaughlin 1967; for review see Pélisson et al. 2010) with the displacement of the fixation target to the periphery being the first and the intra-saccadic displacement being the second step. Although saccade adaptation is typically studied using backward or forward steps of the target, saccades can also adapt if the target is stepped perpendicular to the saccade direction (cross-axis adaptation; e.g. Chen-Harris et al. 2008; Wallman and Fuchs 1998), leading either to changes in saccade gain (saccade amplitude relative to target eccentricity) or changes in saccade direction. Saccade adaptation does not require the target to be stepped consistently, but also occurs if the target is shifted randomly (e.g. Srimal et al. 2008; Havermann and Lappe 2010). However, with random target steps the adaptation of saccades is less conspicuous.

Traditionally, saccade adaptation has been thought of as a reflexive mechanism minimizing the need for corrective saccades or minimizing the retinal error, i.e. the post-saccadic distance between eye and target position. This view has changed over the decades. First, because a variety of studies have meanwhile shown that saccade adaptation is not necessarily reflexive but can be modulated or even caused by more high-level aspects, for example by reward-contingencies or by the behavioral relevance of a target (for reviews see Madelain et al. 2011a; Souto and Schütz 2020). Second, studies have revealed that partly different mechanisms and brain regions are involved when adapting reactive saccades using the double-step paradigm compared to the adaptation of voluntary saccades (Deubel 1995; Gerardin et al. 2012; Panouillères et al. 2014; van Es and Knapen 2019). Third, saccade adaptation can occur in the absence of corrective saccades (Noto and Robinson 2001; Wallman and Fuchs 1998) and the error signal giving rise to saccade adaptation involves an internal prediction of the actual endpoint, not just the factual difference between end point and target location, i.e. retinal error (Bahcall and Kowler 2000; Collins and Wallman 2012; Wong and Shelhamer 2011). Bahcall and Kowler (2000), for example, asked participants to saccade 75% of the distance to a peripherally appearing target that was stepped 20% backward during the saccade. Thus, usage of a retinal error would have predicted forward adaptation. However, saccade amplitude decreased over the course of the experiment which was taken as evidence for the role of prediction in saccade adaptation. Wong and Shelhamer (2011) had retinal and prediction error directly compete with each other in an experiment without explicit instructions. They made use of the fact that saccades are hypometric and typically undershoot the target by 5–10%. In their experiment, the saccade target disappeared during the saccade and appeared after saccade offset, located in-between the saccade endpoint and the original pre-saccadic target location. Therefore, usage of a retinal error would have been consistent with forward and usage of a prediction error with the actually observed backward adaption. The data favoured the latter. Fourth, findings that saccadic adaptation also affects visuospatial perception, specifically the localization of visual targets (Awater et al. 2005; Bahcall and Kowler 1999; Gremmler et al. 2014; Hernandez et al. 2008; Moidell and Bedell 1988; Zimmermann and Lappe 2009) have suggested that adaptation occurs at multiple levels in the visuomotor transformation. Adaptation even occurs for tiny target shifts within the fovea (Havermann et al. 2014), where visual acuity is not in question. This shows that the aim of adaptation is not only to produce good foveal vision but also to maintain consistency between visual and motor representations of space in the brain (Collins et al. 2007a; Zimmermann and Lappe 2016).

Saccade adaptation depends on whether saccades are initiated in response to a peripherally appearing stimulus or voluntarily. Voluntary saccades are internally rather than externally triggered and occur, for example, when scanning a visual scene. It is assumed that adaptation of voluntary and reactive saccades involves at least partly independent mechanisms. Deubel (1995) tested whether the adaptation of voluntary saccades transfers to interleaved reactive saccade and vice versa. He found transfer of adaptation within but not between these two categories. This finding has been replicated by several studies who also showed that the transfer from voluntary to reactive saccades is stronger than the other way round (Alahyane et al. 2007; Collins and Doré-Mazars 2006; Cotti et al. 2007; Kojima et al. 2015). Alahyane et al. (2007) suggested a two-level scheme for adaptation of reactive and voluntary saccades in which both types share a common final pathway but rely on partly independent loci on the previous level. A subsequent study focusing on patients with cerebellar lesions suggested that this previous level might be located in the cerebellum (Alahyane et al. 2008). However, adaptation of these two saccade types also involves differential cortical contributions (Gerardin et al. 2012; Panouillères et al. 2014). Further differences in the adaptation of reactive and voluntary saccades can be found in the time course of learning. Sensorimotor adaptation in general is often described as a dual-state model, i.e. a combination of a fast process that learns but also forgets quickly and a slow process that forgets slowly but also takes more time to learn. Such a dual-state model is able to account for the time-course of learning, recovery as well as re-learning, and can be linked to explicit and implicit learning processes (Ethier et al. 2008; Huberdeau et al. 2015; McDougle et al. 2015; Smith et al. 2006). A recent study directly compared the contribution of the fast and the slow learning process in adaptation of voluntary versus reactive saccades (van Es and Knapen 2019): Whereas fast and slow learning both contributed to adaptation of reactive saccades, adaptation of voluntary saccades was mostly determined by slow learning. The authors concluded that the task demands of voluntary saccade adaptation interfered with explicit learning.

Madelain et al. (2011b) showed that saccade adaptation can be caused by reinforcement learning rather than displacing the target during the saccade. Even if the same saccade vector is repeated multiple times, the amplitude of all saccades will not be identical but will vary from trial to trial. Madelain et al. (2011b) made use of this natural variability in saccade amplitude and reinforced saccades in a pre-defined amplitude range. Across different experiments, they either used an auditory tone or the appearance of the target at the fovea as reinforcer. They found overall changes in saccade amplitude that are comparable with the overall changes found when displacing the target during the saccade. Taken together, although saccade adaptation has classically been considered a low-level mechanism for error-correction that was mainly attributed to the cerebellum, the findings noted above highlight the role of task demands and motivational influences. This parallels adaptation deficits observed in patients with Parkinson’s disease (Abouaf et al. 2012; MacAskill et al. 2002) which suggest that areas other than the cerebellum contribute to the maintenance of saccades. Particularly the involvement of the basal ganglia underpins the susceptibility of saccade adaptation to reward and motivation.

### What renders a saccade target rewarding?

#### The prospect of reward

**Fig. 2:**
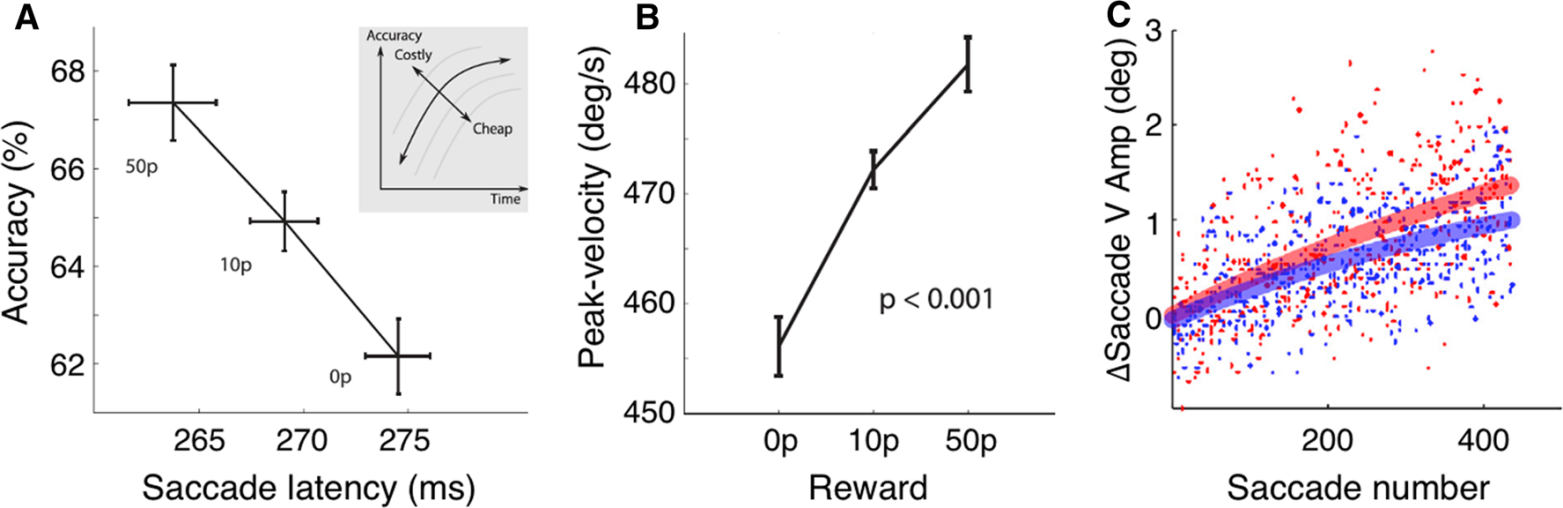
Reward effects on saccadic eye movements. **a** In anticipation of a monetary reward human saccades are more accurate (i.e. fewer erroneous saccades to a distractor) and initiated with a shorter latency as reward increases (50p: 50 pence). The inset shows how this reward effect relates to the speed-accuracy trade-off: Reward increases both, speed and accuracy, thus breaking the typical lawful relationship between the two. **b** Increased saccade peak-velocity with increasing anticipated reward. Error bars denote one standard error of within-participant variability. Panels (**a**, **b**) are adapted from Manohar et al. (2015). **c** Stronger saccade adaptation in monkeys following a food reward (red) compared to unrewarded adaptation (blue). Dots represent data from individual saccades, solid lines denote fitted exponential functions. Panel (**c**) is adapted from Kojima and Soetedjo (2017)

When monkeys anticipate a food reward for an eye movement, changes in oculomotor behavior can be observed in all of the aforementioned metrics: Saccades are more accurate, they are initiated with shorter latencies, higher peak-velocities (Chen et al. 2013; Kawagoe et al. 1998; Platt and Glimcher 1999; Sugrue et al. 2004; Takikawa et al. 2002) and they adapt more rapidly (Fig. 2C; Kojima and Soetedjo 2017). These behavioral changes come along with changes in the basal ganglia (Kawagoe et al. 1998) and the superior colliculus (Ikeda and Hikosaka 2003, 2007). Similar oculomotor changes can be observed in humans expecting a monetary reward (Chen et al. 2014; Clark and Gilchrist 2018; Dunne et al. 2015; Manohar et al. 2015, 2017; Milstein and Dorris 2007). These similarities are not self-evident given both, a potential difference between species and, in particular, a difference between primary and secondary rewards (i.e. food vs money). In humans, different reward types belonging either to primary or secondary rewards activate a common reward circuitry but also selectively recruit brain structures depending on the reward type (for review see Sescousse et al. 2013).

In the study by Milstein and Dorris (2007) participants received a reward for reactive saccades to single targets. Targets could appear left or right from fixation and in every block one of the two locations was associated with a high reward, the other location with a low reward. The authors varied the reward magnitude associated with a target as well as the probability of the target to appear at a given location. Saccade latencies were affected by both, reward magnitude and target probability, but showed a clear negative correlation with expected value, the combination of the two factors, suggesting that reactive saccades are indeed sensitive to expected value. However, this result pattern arises as a consequence of mixing single-target responses with choices among two simultaneously displayed targets (Wolf et al. 2017). When given the choice to decide among two differently valued targets, people are quite reliable in selecting the option with a higher reward. Interleaving choices with reactive saccades delays reactive saccades to the less-valued option due to the choice-induced suppression of the target providing fewer reward. This is reflected in a lateralization of pre-stimulus alpha power and a lowered baseline activity in the LATER model respectively (Heuer et al. 2017; Wolf et al. 2017). The slowing of reactive saccades to the less rewarded option is particularly pronounced if the non-chosen option becomes the target for the subsequent single-target response and depends on the individual preference of one choice target over the other (Wolf and Schütz 2019). Thus, latencies of reactive saccades may be sensitive to the presence or absence of a reward prospect, but not necessarily to different levels of reward magnitude. One explanation for this might be that reactive saccades are mostly initiated too early for differentiated value information to be considered. This might explain why effects of reward magnitude are more prominent in other effectors, e.g. manual responses, that come along with higher reaction times (Clark and Gilchrist 2018; Heuer et al. 2019).

A different picture emerges when multiple potential saccade targets compete, either because the prospect of reward might be more strongly reflected in the rejection of non-desired options, or because of the increase in saccade reaction times caused by the presence of an additional distractor. In an experiment by Manohar et al. (2015) participants fixated one out of three equidistant discs. The other two discs lit up one after the other and participants were instructed to look at the disc being lit second. At the beginning of each trial, it was announced whether correct selection would go along with a high, low or no reward. With increasing reward, saccade selection was faster (earlier saccades as well as higher peak-velocities) and more accurate at the same time (Fig. 2A, B). This modulation by reward was less pronounced in a group of Parkinson patients (Manohar et al. 2015), highlighting the role of dopamine and the basal ganglia for reward-driven oculomotor behavior in humans. The authors concluded that motivation by reward can break the lawful relationship between speed and accuracy by reducing intrinsic neural noise. Earlier and faster saccades in the face of reward are compatible with the view that the motor system is tuned to increase the reward rate, i.e. the amount of reward obtained per time, by increasing movement vigor via dopaminergic activity (Beierholm et al. 2013; Niv et al. 2007).

When looking at the temporal dynamics of oculomotor selection among two competing targets or target regions, then short-latency saccades are typically guided by physical salience whereas long-latency saccades are driven by top-down aspects like the prospect of reward (Ludwig and Gilchrist 2002; Markowitz et al. 2011; Schütz et al. 2012; van Zoest et al. 2004; Wolf and Lappe 2020). This dynamic transition from bottom-up to top-down oculomotor control could either depend on the time it takes to integrate value information into the saccade plan (Schütz et al. 2012) or by the time it takes to inhibit the orienting response caused by the suddenly appearing salient stimulus. Recent evidence supports the latter option (Wolf and Lappe 2020): Saccade endpoints were temporally biased towards salience whenever a salient item suddenly appeared or re-appeared close to a designated and rewarded saccade target. This onset bias was found even when a saccade to the rewarded target had been pre-planned and the onset was fully predictable. Successfully selecting the rewarded target could only be achieved by pre-viewing the salient stimulus in the periphery (Wolf and Lappe 2020). These temporal dynamics share characteristics with the global effect, i.e. the tendency to saccade in-between the designated saccade target and a distractor (Findlay 1982). First, early saccades are biased by the presence of the distractor, long-latency saccades are mostly accurate (Coëffé and O’Regan 1987; McSorley and Findlay 2003; Ottes et al. 1985). This time course might rely on the same oculomotor inhibition responsible for the dynamic changes observed in saccade curvature (van der Stigchel 2010). Second, the global effect can be reduced by the presence of visual information (Arkesteijn et al. 2020, 2018). Third, it occurs independent of whether a saccade is preprogrammed or not (Arkesteijn et al. 2020).

Visual selection in general is not only affected by the immediate prospect of reward but also by the preceding individual selection and reward history, both on a short (e.g. Hickey and van Zoest 2012) and long timescale (e.g. Anderson et al. 2011; Theeuwes and Belopolsky 2012), paralleling the findings of short-term and long-term learning of value in the caudate nucleus (Kim and Hikosaka 2013). These history effects are beyond voluntary control and can even conflict with behavioral goals (Le Pelley et al. 2015; Theeuwes and Belopolsky 2012). In recent years many studies focused on how previously selected targets and reward history bias gaze and attention and this research is comprehensively covered by recent review articles (Anderson 2013; Awh et al. 2012; Bourgeois et al. 2016; Failing and Theeuwes 2018; Le Pelley et al. 2016).

#### Perceptually relevant targets

The modulation of saccade characteristics by the prospect of reward highlights that the oculomotor system is susceptible to motivational influences. Yet, it can be argued that obtaining a monetary reward for an eye movement is an artificial scenario, because the oculomotor systems’ purpose in everyday life is to select parts of the visual field that provide relevant information and reduce visual uncertainty. Bray and Carpenter (2015) compared saccades to targets that provided reliable versus unreliable information about the subsequent target location. They found shorter latencies to informative targets and the changes in latency distributions were consistent with a steeper rate-of-rise in the LATER decision signal. This suggests that if multiple targets compete, the one which is expected to provide more information is more likely to be looked at which is in line with eye movement behavior in natural vision (Land et al. 1999; Rothkopf et al. 2007; Sullivan et al. 2012). These effects might be mediated by neurons in LIP which are sensitive to the expected gain in information that can be obtained by making a saccade to a particular target (Foley et al. 2017; Horan et al. 2019). Moreover, a sustained response to task information first appears in LIP and prefrontal cortex before this information is passed on to other eye movement related areas like FEF (Siegel et al. 2015).

**Fig. 3:**
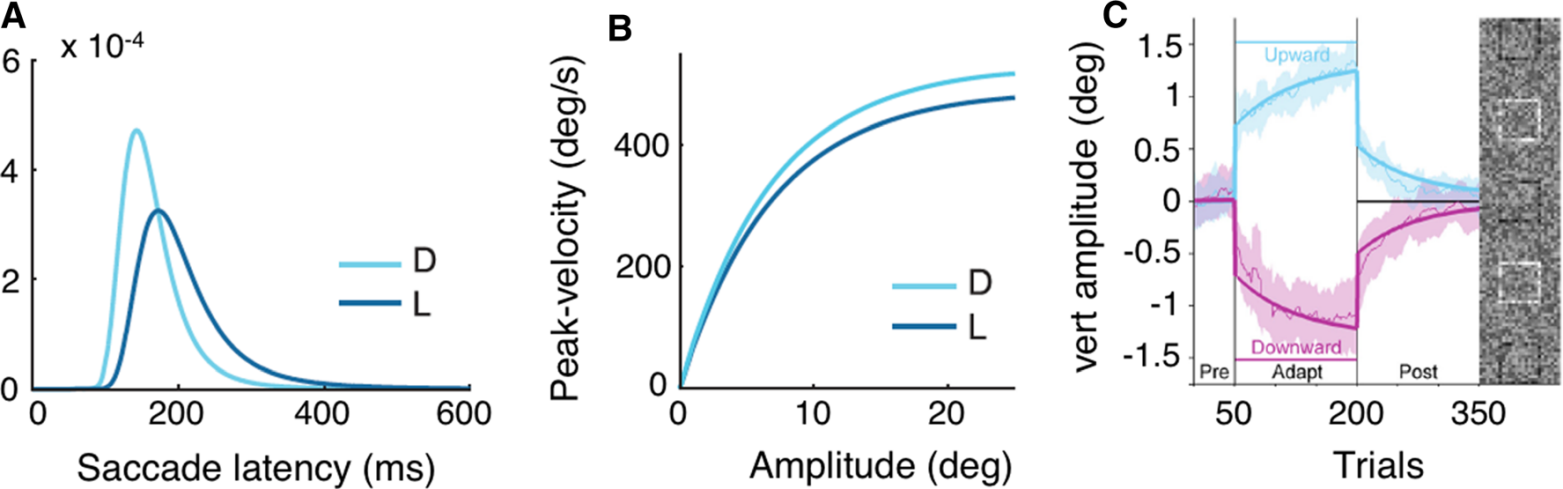
Effects of task-relevance on saccadic eye movements. **a**, **b** When saccades are made to stimuli that also serve as targets for a perceptual task (“D”, light blue), saccades are initiated earlier (A) and with higher peak-velocities (**b**) compared to when participants are merely instructed to saccade to the target as quickly as possible (“L”, dark blue). **a**: Saccade latency distributions derived from fitting the LATER model. Differences in distributions are mostly caused by a steeper rate-of-rise of the decision signal. **b** Exponential fit to the main sequence data. Differences in peak-velocity are particularly prominent for large amplitude saccades. Panels (**a**, **b**) adapted from Bieg et al. (2012). **c** Perceptual tasks affect target selection processes in saccade adaptation. The lines show vertical saccade amplitude over the time course of the experiment. Participants had to saccade to a vertical compound stimulus (shown on the right) and discriminate the opening of one of the elements. During the pre-adaptation and post-adaptation phase, the central element had to be discriminated. During the adaptation phase a vertically eccentric element was task-relevant, causing either upward (light blue) or downward adaptation (purple). The data (thin lines) can be explained by a dual-state model (thick lines) that combines an immediate strategic adjustment and slow gradual learning. Panel (**c**) is adapted from Schütz and Souto (2015)

A variety of studies directly compared saccades to targets that provided task-relevant information with conditions in which saccades were made to irrelevant targets (Bieg et al. 2012; Guyader et al. 2010; Montagnini and Chelazzi 2005; Trottier and Pratt 2005; Wolf and Schütz 2017). Montagnini and Chelazzi (2005) measured saccades to a peripheral target that briefly turned into a letter and could only be discriminated using foveal vision. Critically, the peripheral target turned into the letter after a time period that was determined beforehand by the median individual saccade latency plus saccade duration. As a consequence, participants were only able to discriminate the letter when their latency was below their previously determined median latency, giving rise to perceptual urgency. This perceptual urgency manipulation led to shorter latencies and increased peak-velocities and was reflected in a steeper and less variable rate-of-rise in the LATER model. The same pattern of saccade metrics and a steeper rate-of-rise in the LATER model were obtained by Bieg and colleagues who compared task-related saccades and saccades to uninformative targets without introducing urgency (Fig. 3A, B; Bieg et al. 2012). Importantly, when making saccades to uninformative targets, participants were instructed to look at the target as quickly as possible. The observation of earlier saccades to task-relevant targets also holds when the periphery provides more task-relevant information than the post-saccadic foveal image and the saccade thus comes along with an increase rather than a reduction of perceptual uncertainty (Wolf and Schütz 2017), suggesting that foveation of relevant targets represents an overlearned and rigid behavior that can result in suboptimal information sampling (Clarke and Hunt 2016; Morvan and Maloney 2012). Such a preference for foveal sampling (Gloriani and Schütz 2019) might result from the lifetime experience that most relevant information can be obtained by directly looking at a target.

The notion that participants prefer to obtain foveal information about targets rather than relying on their peripheral vision is additionally supported by experiments in which multiple relevant locations compete for selection (Clarke and Hunt 2016; Morvan and Maloney 2012; Nowakowska et al. 2017; Renninger et al. 2007; Tsank and Eckstein 2017). In an experiment by Morvan and Maloney (2012), participants could saccade to a compound of three horizontally spaced squares in order to localize a small dot that appeared after the saccade. The dot could appear either in the most left or the most right square and participants had to indicate whether it was located within the upper or lower half of a square. Across trials, the distance between squares was varied. Depending on this distance, the optimal saccade strategy would have been to either saccade to the task-irrelevant central square and monitor the other two locations using peripheral vision or, if the squares were further apart, to guess and select one of the outer squares. Critically, to sample perceptual information efficiently, participants would have switched from a center-strategy to a side-strategy with increasing square distance, yet they did not systematically adjust their saccade endpoints and thus their saccade strategy. These findings have been replicated by Clarke and Hunt (2016) who also showed that this inefficiency is not restricted to the oculomotor domain but may arise due to the participants’ uncertainty about their own perceptual, motor or memory abilities. Although other studies have reported that human observers may exploit their uncertainty across the retina optimally (Najemnik and Geisler 2005), saccade endpoint selection can in many cases be described as reducing uncertainty at the selected endpoint rather than the global image (Renninger et al. 2007). Such a foveal sampling strategy might be disadvantageous in selected laboratory settings, but not in (more) natural vision where eye movements do not come at a high perceptual or energetic cost as movements of other effectors (Solman and Kingstone 2014). Thus, although characterized as sub-optimal in laboratory studies, additional fixations due to consecutive foveations might be used to overcome memory constraints, to visually explore a scene or to increase perceptual confidence (Hayhoe et al. 2003; Land et al. 1999; Nowakowska et al. 2017).

Making a specific element of a target task-relevant can elicit saccade adaptation (Fig. 3C; Schütz et al. 2014; Schütz and Souto 2015; for review see Souto and Schütz 2020). Schütz et al. (2014) had participants saccade to an either horizontally or vertically stretched compound stimulus consisting of multiple characters. When one of the characters became task-relevant and had to be discriminated after the saccade, saccades adapted in the direction of this task-relevant character. Task-elicited adaptation could be best captured by a model that combined an immediate explicit adjustment in addition to the slow, implicit learning from one trial to the next (Schütz et al. 2014). Like classical double-step adaptation, task-elicited adaptation changes saccade amplitudes by a comparable magnitude, transfers to reactive saccades and affects saccade curvature (Schütz et al. 2014) as does cross-axis adaptation with an intra-saccadic step (Chen-Harris et al. 2008). A perceptual task affects saccade adaptation via target selection processes (Collins et al. 2007b; Schütz and Souto 2015; Wolf et al. 2019). When one pre-saccadic target splits into two post-saccadic targets which compete for selection, then saccades normally adapt towards the more salient target. This default selection mode can be overcome when a perceptual task renders the less salient target more informative (Wolf et al. 2019).

Studies on task-irrelevant perceptual learning suggest that (foveally) obtaining task-relevant information may provide an internal reward for the visual system (Seitz and Watanabe 2003, 2005). In an experiment by Seitz and Watanabe (2003) participants had to foveally discriminate light-grey target letters presented in a stream of otherwise dark-grey distractor letters. Simultaneously, a task-irrelevant random-dot kinematogram was shown surrounding the letter location. A certain percentage of dots were moving in the same direction, yet this percentage was set such that no coherent motion direction could be perceived. Critically, the same subliminal motion direction was paired with the foveal presentation of target letters whereas other motion directions were shown equally often but paired with the foveally presented distractor letters. After extensive training, participants showed a benefit in motion discrimination that was exclusive for the motion direction paired with target letters. This was interpreted as subliminal learning not being a passive process that is determined by sheer exposure, but that successful target recognition triggers an internal reinforcement signal that causes learning of task-relevant as well as task-irrelevant signals (Seitz and Watanabe 2003, 2005). Task-irrelevant perceptual learning does not necessarily require an internal task-related reinforcement signal but can also arise as a consequence of externally reinforcing one of the foveally presented letters in the stream (Seitz et al. 2009). This relationship between external or internal reward signals and task-irrelevant perceptual learning was suggested to be mediated by neuromodulatory factors like acetylcholine and dopamine (Roelfsema et al. 2010).

#### Intrinsically valuable stimuli

When we encounter a reward, it first impinges on us through the sensory input. Empirical findings suggest a close link between vision on the one hand and reward-related activity in the dopaminergic midbrain and connected cortical areas on the other hand (Hickey et al. 2010; Hickey and Peelen 2015; Noudoost and Moore 2011). Reward-related modulations can even be found in early visual areas that are typically considered to represent low-level properties of the target (Serences 2008). In an fMRI study by Hickey and Peelen (2015), participants had to report whether exemplars of a cued category were present or not. One of the three used categories was associated with a high reward and exemplars of other categories could be present as distractors. The encoding of these reward-related targets was enhanced in visual areas. In contrast, the encoding of reward-related distractors was reduced although their presence increased reaction times, highlighting that reward does not per se enhance the representation of reward-related objects, but that they need to be actively suppressed (Gaspelin et al. 2015; Hickey and Peelen 2015). This active suppression of salient or reward-related distractors was related to dopaminergic midbrain activity (Hickey and Peelen 2015) and is presumably also reflected in the time course of saccade endpoints when a distractor and a target compete for overt visual selection (Arkesteijn et al. 2020; Coëffé and O’Regan 1987; Ottes et al. 1985; Wolf and Lappe 2020).

This tight link between vision and reward is additionally underpinned by studies showing that the sensory consequences of saccades, i.e. post-saccadic vision of the target itself, appear to act like a reward for the visual system (Collins 2012; Madelain et al. 2011b; Meermeier et al. 2016, 2017b). For example, Collins (2012) showed that post-saccadic foveal feedback (i.e. seeing the saccade target) compared to seeing a blank screen after the saccade led to reduced saccade latencies and improved resistance against distractors. Furthermore, the post-saccadic target view can also be used as a reinforcer for saccade adaptation in the absence of an error signal. Madelain et al. (2011b) presented the target without retinal error on the fovea after the saccade whenever the saccade happened to be close to a goal amplitude and presented a blank screen instead when the saccade did not match the goal amplitude. This, in time, resulted in saccades more closely matching the goal amplitude. The observation that the post-saccadic target can act as a reinforcer is particularly noteworthy given the role of dopaminergic midbrain structures for oculomotor learning (Abouaf et al. 2012; MacAskill et al. 2002) and motivational contributions to motor control in general (Manohar et al. 2015; Mazzoni et al. 2007). Reinforcing saccades by their perceptual consequences is not restricted to seeing any versus no post-saccadic target in the fovea, but also by showing a target versus showing a distractor (Vullings and Madelain 2019). These reinforcing capabilities of seeing a target compared to seeing nothing or seeing a distractor might therefore be mediated by the same intrinsic reward signals that are considered causal for task-irrelevant perceptual learning (Roelfsema et al. 2010; Seitz and Watanabe 2003, 2005).

**Fig. 4:**
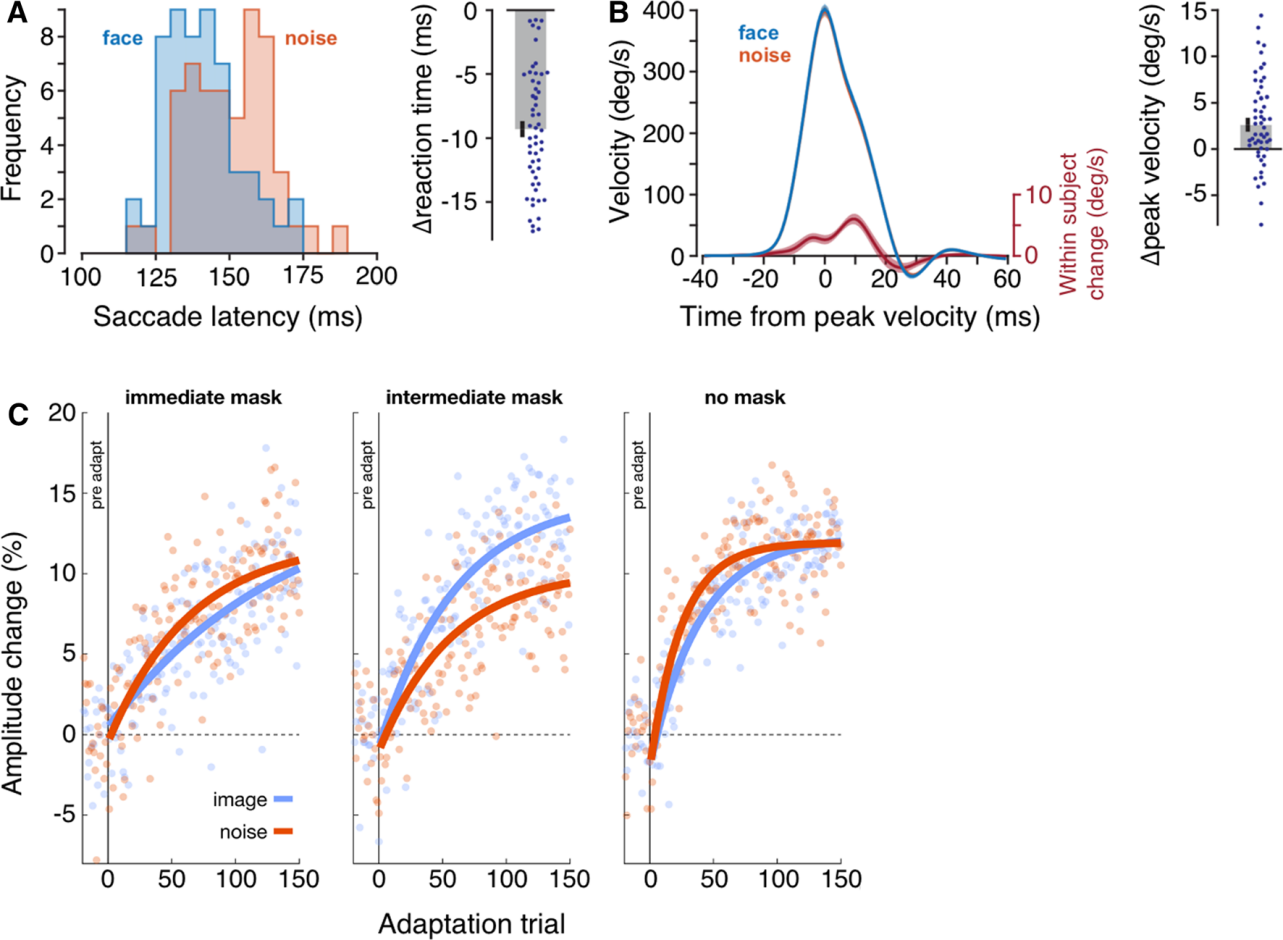
Effects of image content on saccadic eye movements. **a**, **b** Saccades to intrinsically valuable stimuli like faces are initiated earlier and with higher peak-velocities compared to meaningless noise stimuli. **a** Distribution of mean reaction times for the two image categories (left panel) as well as the distribution of mean individual latency differences (right panel). **b** Left panel: Velocity profile for saccades towards faces (blue) and saccades towards meaningless noise patches (orange; mostly hidden). The red line denotes the within-participant change in the velocity profiles. Note that differences are more pronounced in the deceleration than the acceleration phase (see also Kojima and Soetedjo, 2017). Right panel: Distribution of mean individual peak-velocity differences. **a**, **b** are adapted from Sedaghat-Nejad et al. (2019). **c** Saccade adaptation to meaningful images (blue) versus meaningless noise patches (orange). Saccade adaptation towards meaningful images was more complete when images were masked 200 ms after saccade offset (intermediate mask, center panel), allowing a short glimpse of the target but no time for corrective saccades, compared to conditions in which images were masked immediately (left panel) or not at all (right panel). Dots denote mean values across individuals for a given trial number, solid lines are fitted exponentials. **c** is adapted from Meermeier et al. (2016)

Certain categories of stimuli are particularly successful in facilitating saccades. Saccadic selection of animals (Kirchner and Thorpe 2006) or especially saccadic selection of human faces (Crouzet et al. 2010; Kauffmann et al. 2019; Sedaghat-Nejad et al. 2019) can be extremely rapid (Fig. 4A). Although faces have characteristic low- and mid-level features, particularly with regard to amplitude spectrum (Ruiz-Soler and Beltran 2006) that contribute to rapid saccade selection (Crouzet and Thorpe 2011; Honey et al. 2008), the special status of faces for oculomotor behavior goes beyond the low-level image-computable salience as it would be captured by a saliency map (End and Gamer 2017; Marat et al. 2013; Nummenmaa et al. 2009). Dissociating the more cognitive, high-level effects from low-level effects can be achieved by presenting stimuli either only after the saccade (Xu-Wilson et al. 2009), by equating or exchanging low-level properties of various targets (Crouzet and Thorpe 2011; Meermeier et al. 2016; Willenbockel et al. 2010) or by using stimuli that are meaningless or meaningful depending on the participants’ prior knowledge (Teufel et al. 2018).

What is it that makes it special to look at a face? Unlike other stimulus categories, faces are processed holistically (Tanaka and Farah 1993) and a whole neural architecture is devoted to their analysis (for review see Tsao and Livingstone 2008). Inverting a face interferes with its holistic processing (for review see Maurer et al. 2002), rendering inverted faces a further suitable control condition in various cases. The preference to look at images that carry social information is not restricted to humans but can be found in other primates (Deaner et al. 2005; Klein et al. 2008). Imaging studies in humans further suggested that cortical representations of object categories are not uniform across the visual field, but show an eccentricity bias, i.e. faces are biased towards the representation of the central visual field whereas buildings are biased towards the periphery (Levy et al. 2001). This central visual field bias also exists for other stimulus categories, like words, that strongly depend on foveal processing (Hasson et al. 2002). Yet, although the foveal bias for certain stimuli like faces is under debate (Rousselet et al. 2005) and although the exact mechanisms underlying rapid saccades to selected stimulus categories are not fully understood, the aforementioned studies show the importance of foveal processing for these targets, particularly as discrimination performance decreases rapidly the further a face is placed in the periphery (Kreichman et al. 2020).

This importance of foveal processing for particular targets like faces is also mirrored in various studies on saccadic eye movements. These studies corroborate the view that the oculomotor system is optimized to rapidly provide clear foveal vision of targets that are either externally rendered relevant, for example by means of reward, or that are inherently perceptually relevant. In line with the findings on externally relevant targets, this is achieved by the same oculomotor signature (Fig. 4): reduced saccade latencies (Crouzet et al. 2010; Kauffmann et al. 2019; Rothkirch et al. 2013; Sedaghat-Nejad et al. 2019), increased peak-velocities (Sedaghat-Nejad et al. 2019; Xu-Wilson et al. 2009) and more strongly modulated saccade gain in order to have the target accurately placed on the retina (Meermeier et al. 2016, 2017b).

Xu-Wilson et al. (2009) recorded reactive saccades to a dot stimulus. After the saccade, an image was displayed at the post-saccadic position, either a face, an inverted face, an object or pixel noise. Approximately one second before the dot appeared, the same face could be shortly pre-viewed in the periphery and disappeared so that participants knew in advance which image type they can expect when making a saccade to the dot. When expecting to see a face, saccades were characterized by a higher vigor, i.e. they tended to have higher peak-velocities, shorter durations, higher peak accelerations and lower peak decelerations. These findings were explained in terms of an optimal control framework that balances two costs (Shadmehr et al. 2010; Xu-Wilson et al. 2009): a cost associated with a stronger motor command and a second cost associated with the passing of time. The first cost increases with the motor command leading to more endpoint variability (Harris and Wolpert 1998) and thus favors slower movements. The second cost is related to an intrinsic value of the target that is temporally discounted over time and thus favors faster movements to ensure an earlier foveation, particularly if the target has a high intrinsic value. The incorporation of value and its temporal discounting over time in motor control can explain why deficits in brain areas devoted to the processing of reward (e.g. the basal ganglia in Parkinson’s disease) lead to changes in motor control that appear to be related to implicit motivation rather than motor abilities (Mazzoni et al. 2007). It can explain the typical oculomotor signature when saccades are made in anticipation of reward (Manohar et al. 2015; Takikawa et al. 2002) or made to targets that are relevant for perception (Bieg et al. 2012; Montagnini and Chelazzi 2005). Temporally discounting the reward of seeing a target is reflected in saccade peak-velocities and durations (Haith et al. 2012). These oculomotor markers vary substantially across individuals but correlate with an individual’s discounting of reward in decision making, thus the preference of immediate smaller rewards over larger rewards obtained at a later point in time (Choi et al. 2014), suggesting that (oculo)motor control and cognitive decisions may partly share the same valuation process and the same cost of time.

The intrinsic value of a target is also reflected in changes in saccade gain and these changes were shown to be particularly dependent on post-saccadic foveal processing of the target (Meermeier et al. 2016, 2017b). Based on the observation that gaze is naturally drawn towards faces and bodies, Meermeier et al. (2016) studied saccade adaptation towards images of attractive women and compared this with saccade adaptation towards meaningless noise patterns (Fig. 4C). Critically, images were either (i) not masked, (ii) masked immediately, such that no post-saccadic vision of the target was possible, or (iii) masked 200 ms after saccade onset, thus allowing a short post-saccadic glimpse without the possibility for the execution of corrective saccades. If oculomotor learning was modulated by the peripheral preview of an intrinsically relevant target, saccade adaptation to relevant targets would be expected to be more complete in all three masking conditions. However, this was only the case when a short post-saccadic glimpse of the target was possible, highlighting that oculomotor learning was not affected by post-saccadic foveal vision per se, but by the necessity to foveate the target with the primary saccade (Meermeier et al. 2016, 2017b).

Two frequent observations in the processing of rewards are that (i) novel options are assigned higher values which is reflected in the dopaminergic activity in the basal ganglia (Bunzeck and Düzel 2006; Ljungberg et al. 1992; Wittmann et al. 2008) and that (ii) dopamine neurons encode a reward prediction error (for review see Schultz et al. 1997). Thus, they preferably respond when a reward is unexpectedly large. Recent studies have shown that both of these observations are also mirrored in the saccade system. First, saccade adaptation is more complete when a novel image of a human figure is shown every trial compared to repeatedly showing the same image (Meermeier et al. 2017a). Second, reward prediction error (RPE) has recently been shown to affect saccade latencies (Sedaghat-Nejad et al. 2019). In the experiment by Sedaghat-Nejad et al. (2019), participants had to make a sequence of two saccades. The first saccade was made in response to either a face or a visual noise image. Results of primary saccades replicated the finding of shorter latencies and higher peak-velocities towards faces (Fig. 4). During this primary saccade both, the content of the image as well as its position could be changed. As a consequence, participants had to make a second saccade to foveate the image. Importantly, images could either change from a low-value noise stimulus to a high-value face stimulus (implying a positive RPE) or the other way around from a face to a noise stimulus (implying a negative RPE). Conditions in which only the location of the target was changed but not its identity served as control. Following a positive RPE, secondary saccades were initiated earlier compared to the control condition in which a face was also shown at the primary location, whereas the opposite was true for secondary saccades following a negative RPE. Thus, the higher the reward prediction error, the earlier a target was foveally inspected.

The stimuli used in oculomotor studies of intrinsic reward have typically been images of faces, bodies, or animals. It is currently not known whether other image categories provide similar intrinsic value, which aspects of an image are responsible for its rewarding properties, or whether images in general are seen as rewarding. However, initial experiments with images that acquired value through second-order conditioning revealed that primary image content is the main rewarding aspect for saccadic adaptation (Meermeier et al. 2017b). Images of written words or images taken from rewarding configurations of tiles in a popular computer game did not produce the enhancement of saccadic adaptation seen with images of faces. Thus, depending on their content (e.g. faces) images may constitute a primary visual reward as reflected in oculomotor learning.

## Conclusions and future directions

In this review, we summarized how the control of saccadic eye movements is affected by higher-level mechanisms, particularly by reward, task-relevance, and image content, and we emphasized that foveal vision of the target can constitute an internal reward that can be reflected in oculomotor behavior. Beforehand, we laid out which parameters of saccadic eye movements can be used to study cognitive influences and how these influences might be manifested in the underlying neural circuit.

We stressed that saccades to targets that either provide reward, are task-relevant or are intrinsically valuable all share a similar oculomotor signature: (i) shorter latencies, (ii) higher peak-velocities and (iii) faster or more complete saccade adaptation. However, this does not necessarily imply that identical mechanisms are involved. Whereas the anticipation of reward is sufficient to decrease latencies and increase peak-velocities (Manohar et al. 2015), earlier and more vigorous saccades to faces or other intrinsically valuable targets might be driven by peripheral preview of the target image. Thus, changes in oculomotor behavior due to reward anticipation might rely on top-down signals originating in frontal areas, whereas saccades to intrinsically valuable stimuli might require specific bottom-up signals that can further be modulated, for example, by the presence of a reward prediction error (Sedaghat-Nejad et al. 2019). Increased efficiency of saccadic adaptation, on the other hand, requires a view the post-saccadic image and peripheral preview is not sufficient (Meermeier et al. 2016).

Distinguishing different possible mechanisms given similar behavioral output remains an open task for future work. Even in the study by Xu-Wilson et al. (2009) where saccades were made in response to appearing dots, images were shown beforehand at the designated location for a brief time. The motor program could thus have been pre-programmed and executed at a later time point with the same or similar kinematics. Perceptually, pre-saccadic peripheral face information is used for post-saccadic face processing (Buonocore et al. 2020), highlighting the peripherally inspected content is processed. Yet, there is contradictory evidence that, for example, the emotional valence of a face is reflected in saccades towards that face (e.g. Kulke 2019; Nummenmaa et al. 2009), possibly because saccades to faces often occur too early for higher-level image content to exert an influence. The fact that any error signal driving saccade adaptation can only be evaluated after the saccade and thus provides more time for visual processing, might qualify saccade adaptation as a more sensitive and adjustable tool for measuring for the intrinsic value of images.
